# The fecal presence of enterotoxin and F4 genes as an indicator of efficacy of treatment with colistin sulfate in pigs

**DOI:** 10.1186/s12866-016-0915-0

**Published:** 2017-01-05

**Authors:** Mohamed Rhouma, John Morris Fairbrother, William Thériault, Francis Beaudry, Nadia Bergeron, Sylvette Laurent-Lewandowski, Ann Letellier

**Affiliations:** 1 Chaire de recherche industrielle du CRSNG en salubrité des viandes (CRSV), Faculté de médecine vétérinaire – Université de Montréal, 3200 rue Sicotte, Saint-Hyacinthe, QC J2S 7C6 Canada; 2 Groupe de recherche et d’enseignement en salubrité alimentaire (GRESA), Faculté de médecine vétérinaire – Université de Montréal, 3200 rue Sicotte, Saint-Hyacinthe, QC J2S 7C6 Canada; 3 Centre de recherche en infectiologie porcine et avicole (CRIPA), Faculté de médecine vétérinaire – Université de Montréal, 3200 rue Sicotte, Saint-Hyacinthe, QC J2S 7C6 Canada; 4 Groupe de recherche en pharmacologie animale du Québec (GREPAQ), Faculté de médecine vétérinaire – Université de Montréal, 3200 rue Sicotte, Saint-Hyacinthe, QC J2S 7C6 Canada; 5 OIE Reference Laboratory for Escherichia coli (EcL), Faculté de médecine vétérinaire – Université de Montréal, 3200 rue Sicotte, Saint-Hyacinthe, QC J2S 7C6 Canada

**Keywords:** ETEC, Virulence gene, Fecal, Colistin sulfate, Diarrhea, Pigs

## Abstract

**Background:**

Enterotoxigenic *Escherichia coli* (ETEC) strains producing multiple enterotoxins are important causes of post-weaning diarrhea (PWD) in pigs. The aim of the present study was to investigate the fecal presence of ETEC enterotoxin as well as F4 and F18 genes as an indicator of colistin sulfate (CS) efficacy for treatment of PWD in pigs. Forty-eight piglets were weaned at the age of 21 days, and were divided into four groups: challenged treated, challenged untreated, unchallenged treated, and unchallenged untreated. Challenge was performed using 10^9^ CFU of an ETEC: F4 strain, and treatment was conducted using oral CS at the dose of 50,000 IU/kg. The fecal presence of genes encoding for STa, STb, LT, F4 and F18 was detected using PCR.

**Results:**

The PCR amplification of ETEC virulence genes showed that nearly 100% of pigs excreted genes encoding for STa and STb toxins in the feces before the challenge. These genes, in the absence of the gene encoding F4, were considered as a marker for F4-negative ETEC. One day after ETEC: F4 oral challenge pigs in the two challenged groups excreted the genes encoding LT and F4 in the feces. These genes were considered as a marker for F4-positive ETEC. However, the gene encoding F18 was not detected in any fecal samples of the 4 groups throughout the experiment. After only 3 days of successive oral treatment with CS, a significant reduction in both the F4-positive and negative ETEC populations was observed in the challenged treated group compared to the challenged untreated group (*p <* 0.0001).

**Conclusions:**

Our study is among the first to report that under controlled farming conditions, oral CS treatment had a significant effect on both fecal F4-positive and F4-negative ETEC in pigs. However, CS clinical efficiency was correlated with non-detection of F4-positive ETEC in the feces. Furthermore the fecal presence of F4-negative ETEC was not associated with clinical symptoms of post-weaning diarrhea in pigs.

**Electronic supplementary material:**

The online version of this article (doi:10.1186/s12866-016-0915-0) contains supplementary material, which is available to authorized users.

## Background

Post-weaning diarrhea (PWD) is an economically important disease in pigs due to financial losses as a result of mortality, morbidity, diarrhea, reduced growth performance, and medication costs [1, 2]. This disease is usually associated with the proliferation of one or more strains of Enterotoxigenic *Escherichia coli* (ETEC) in the pig gastrointestinal tract [2]. ETEC strains are characterized by the production of enterotoxins and adhesins, both essential for disease development [3]. Enterotoxins produced by ETEC may be heat stable (STa, STb or enteroaggregative *E. coli* heat stable enterotoxin 1 [EAST1]) or heat labile (LT). In pigs, the most frequently observed fimbrial adhesins of ETEC are K88 (F4), F18, K99 (F5), 987P (F6), and F41 [3]. F4-positive ETEC (ETEC: F4) infections represent the major cause of PWD in pigs worldwide [4, 5]. Furthermore, the most predominant serovirotypes of ETEC associated with PWD in pigs are O149: LT: STb: F4 and O149: LT: STa: STb [6]. The diagnosis of PWD in pigs is based on clinical signs, microscopic lesions and bacteriological testing [3]. Bacteriological tests remain the most effective method to confirm the etiology of PWD, and to assess the effectiveness of antimicrobials used in its treatment. Determination of ETEC virulence genes is the most reliable method to identify the presence of pathogenic *E. coli* associated with PWD [4]. Colistin sulfate (CS), a cationic antimicrobial peptide, is one of the most frequently used antibiotics for the treatment of PWD [7, 8], being mostly used per os, at a recommended dose of 50,000 IU/kg body weight (bw) every 12 h for a period of 3 to 5 consecutive days [9]. However, with the increase rate of CS resistance *E. coli* in pigs [9], the monitoring of the therapeutic efficacy of CS appears very important. The aim of the present study was to examine the fecal presence of ETEC enterotoxin as well as F4 and F18 genes in an experimental infection model as an indicator of the effectiveness of CS oral treatment to control the ETEC population in PWD in pigs.

## Methods

The experimental protocol was reviewed and approved by the Institutional Animal Care and Use Committee of the Faculty of Veterinary Medicine (FVM) of the Université de Montréal and it was performed in accordance with the guidelines of the Canadian Council on Animal Care (CCAC).

### Animals and experimental design

The present study was conducted as part of a project designed to assess the pharmacokinetic of CS during the treatment of PWD and its effect on the exacerbation of *E. coli* resistance in pigs [10]*.* Briefly, 48 Duroc-Yorkshire-Landrace pigs, obtained from 11 different litters, were selected at 4 days of age for the presence of the F4 receptor gene by PCR-RFLP as previously described [11].

After weaning (21 days), pigs were randomly divided into four groups of 12 pigs each: challenged treated (originated from 7 litters), challenged untreated (originated from 8 litters), unchallenged treated (originated from 5 litters), and unchallenged untreated (originated from 6 litters). Animals were fed a standard non-medicated rations for post-weaning pigs and had unlimited access to feed and water throughout the experiment.

After 1 week of acclimatization (28 days), pigs in the challenged groups were orally gavaged with 10^9^ CFU of ETEC: F4 strain ECL8559A (O149: LT: STa: STb: F4: Nal^R^) kindly provided by the Reference Laboratory for *Escherichia coli* (EcL, Faculty of Veterinary Medicine from the Université de Montréal) as described previously [10]. The day of the challenge corresponds to d0 in our experimentation.

Colistin sulfate (Bond & Beaulac Inc., QC, Canada) was administered by gavage to the challenged/treated and unchallenged/treated groups, 1 day after the challenge, at a dose of 50,000 IU/kg bw twice a day for 5 successive days.

The rectal body temperature was monitored daily using a digital thermometer. The severity of diarrhea was assessed visually by using a fecal consistency scoring (0, normal; 1, soft feces; 2, mild diarrhea; 3, semi-liquid diarrhea and 4, liquid diarrhea) as previously described [10].

### Fecal sampling and microbiological analysis

Fresh fecal samples were obtained from pigs using pre-weighed sterile rectal swabs (Puritan Medical Products, Guilford, Maine, USA). Sampling of fecal material was performed 1 day before (d-1) and 1, 4, 8, 13, 36 days after the oral challenge.

Fecal swabs were diluted 1:10 in buffered peptone water solution (BPW) and were incubated at 37 °C overnight. A volume of 500 μL of this enrichment was placed in a 4.5 mL of Luria-Bertani (LB) broth and incubated at 37 °C overnight. One millilitre (in duplicate) of each sample was stored at −80 °C for subsequent analysis. Rectal temperatures and diarrhea scores were taken at the same time as the fecal samples.

### DNA extraction and PCR procedure

Fecal presence of genes encoding ETEC virulence factors STa, STb, LT, F4 and F18 was evaluated using PCR as previously described [12]. DNA was extracted by heat lysis. Briefly, 1 mL of each sample was pelleted by centrifugation at 11,750 g for 5 min and 1 mL of Phosphate Buffered Saline (Becton Dickinson and Company, Sparks, MD, USA) was added; samples were pelleted by centrifugation at 11,750 g for 2 min and 500 μL of sterile Milli-Q water was added. Tubes were boiled for 10 min and immediately placed on ice. The boiled cell suspensions were centrifuged at 11,750 g for 2 min, and the supernatants were used for PCR. The genes encoding STa, STb, LT, F4 were detected by multiplex PCR using published primers [13, 14]. Multiplex PCR positive and negative controls were ECL8559 [15], and *Listeria monocytogenes* of porcine origin respectively [16]. However, gene encoding F18 was detected by uniplex PCR using published primers [13], and the PCR positive control for this gene was ECL1033.

PCR procedures were performed according to a protocol of the EcL, available at http://www.apzec.ca/en/APZEC/Protocols/APZEC_PCR_en.aspx. The PCR reactions were performed in a 25 μL volume and comprised 2 μL of MgSO_4_ (20 m*M*), 2.5 μL dNTP (2 m*M*), 2.5 μL of Taq buffer (10×), with 1.25 μL of F18 primers (10 μ*M*) for the uniplex PCR, and 1 μL of STa and LT primers (5 μ*M* and 10 μ*M* respectively), 1.25 μL of STb and F4 primers (10 μ*M* each) for the multiplex PCR, with 1U Taq DNA polymerase (Bio Basic Inc., ON, Canada), and 5 μL of the DNA sample for both PCR. Sterile water was used to bring the final reaction volume to 25 μL. After amplification, a 10 μL aliquot was submitted to electrophoresis in a 1.8% agarose gel stained with SYBR*®* Safe (Invitrogen*,* Burlington, ON, Canada). Amplification products were visualized and photographed under UV illumination.

### Statistical analysis

Percentage of pigs shedding each virulence gene (number of positive pigs/total number of pigs) for each sampling time in 4 groups was analyzed with exact chi-square at each time-period.

Statistical analyses were carried out with SAS v.9.4. (Cary, N.C.). Rectal temperature was analyzed with repeated-measures ANOVA, with time as a within subject factor and group as the between-subject factor. Ordinal diarrhea scores were analyzed with the Cochran-Mantel-Haenszel test at each time-period. The level of statistical significance was set at *p* < 0.05 for all analyses.

## Results

**Fig. 1:**
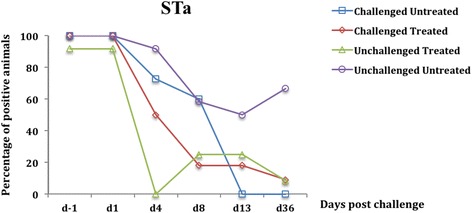
Percentage of fecal presence of the gene encoding STa enterotoxin in weaned pigs challenged or not with ETEC: F4. Challenge was performed at d0 and treatment with colistin sulfate at the dose of 50,000 IU/kg was started at d1 (24 h post challenge) and administered twice daily for 5 days. At d4 a significant reduction in the fecal presence of the gene encoding STa was found in the unchallenged treated group compared to the challenged untreated and the unchallenged untreated groups (*p <* 0.0001). At d36, the fecal presence of the gene encoding STa was statistically lower in the challenged untreated group compared with the unchallenged untreated group (*p* < 0.001). The percentage was calculated by dividing the number of positive pigs by the total number of pigs in each group

**Fig. 2:**
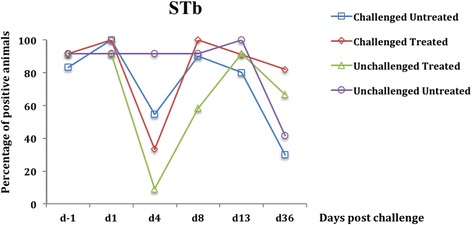
Percentage of fecal presence of the gene encoding STb enterotoxin in weaned pigs challenged or not with ETEC: F4. Challenge was performed at d0 and treatment with colistin sulfate at the dose of 50,000 IU/kg was started at d1 (24 h post challenge) and administered twice daily for 5 days. At d4 a significant reduction in the fecal presence of the gene encoding STb was found in the unchallenged treated group compared to the unchallenged untreated group (*p <* 0.001). The percentage was calculated by dividing the number of positive pigs by the total number of pigs in each group

**Fig. 3:**
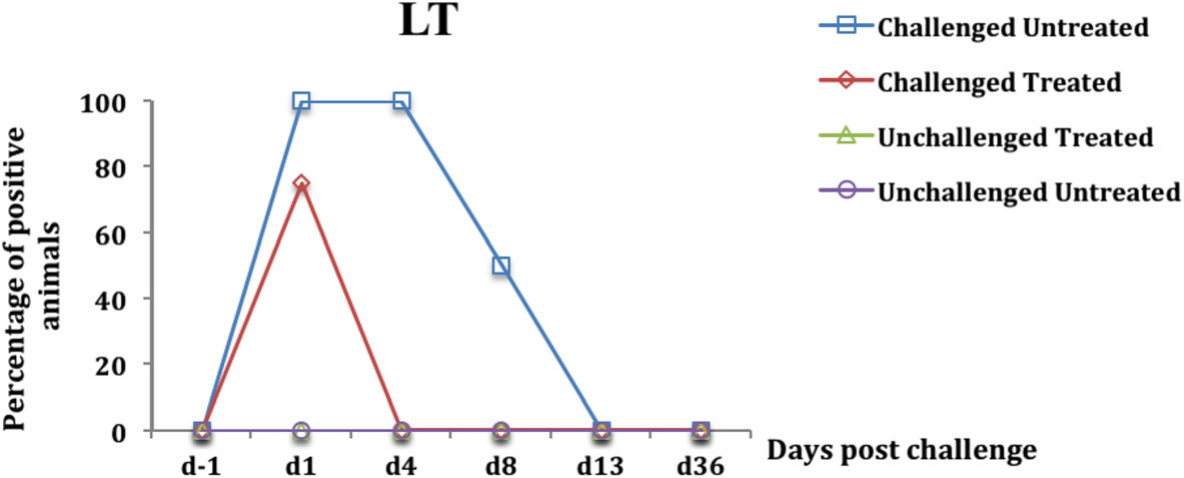
Percentage of fecal presence of the gene encoding LT enterotoxin in weaned pigs challenged or not with ETEC: F4. Challenge was performed at d0 and treatment with colistin sulfate at the dose of 50,000 IU/kg was started at d1 (24 h post challenge) and administered twice daily for 5 days. At d4 a significant reduction in the fecal presence of the gene encoding LT was found in the challenged treated group compared to the challenged untreated group (*p <* 0.0001). The percentage was calculated by dividing the number of positive pigs by the total number of pigs in each group

**Fig. 4:**
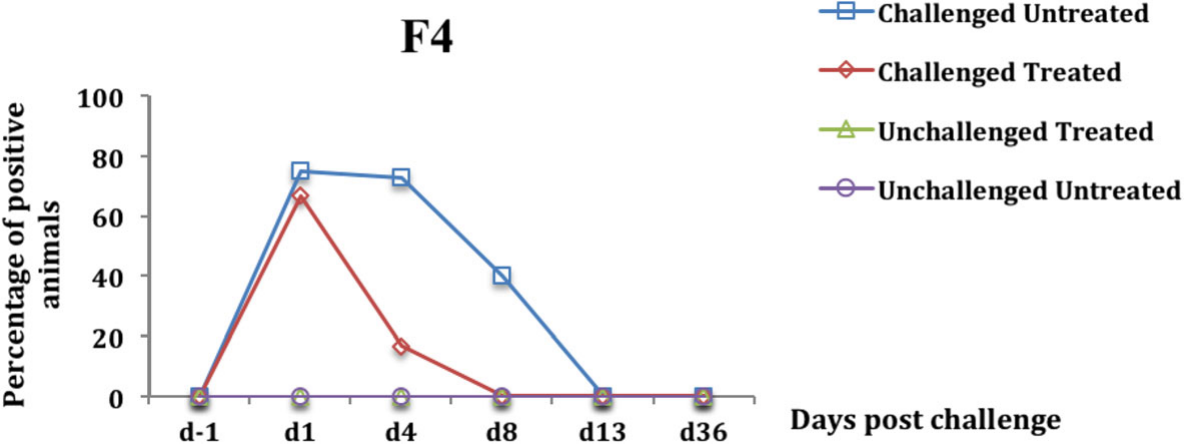
Percentage of fecal presence of the gene encoding F4 in weaned pigs challenged or not with ETEC: F4. Challenge was performed at d0 and treatment with colistin sulfate at the dose of 50,000 IU/kg was started at d1 (24 h post challenge) and administered twice daily for 5 days. The percentage was calculated by dividing the number of positive pigs by the total number of pigs in each group

Prior to the bacterial challenge (d-1), none of the pigs in any of the 4 groups showed signs of diarrhea or anorexia. The PCR amplification of ETEC virulence genes showed that nearly 100% of pigs (28 days) had a fecal presence of genes encoding for STa and STb toxins in the feces before the challenge (d-1) (Figs. 1 and 2), whereas no pig had a fecal presence of genes encoding LT or F4 (Figs. 3 and 4). This finding indicated that all clinically healthy pigs used in this study were infected at weaning with STa- and/or STb-positive *E. coli,* which we refer to as putative F4-negative ETEC isolates. In addition, genes encoding LT and F4 were not detected in any fecal samples in the unchallenged groups throughout the experiment (Figs. 3 and 4). Likewise, genes encoding for F18 virulence factor were not detected in any fecal samples of either challenged or unchallenged groups throughout the experiment.

One day after ETEC: F4 oral challenge (d1), pigs of the two challenge groups excreted the genes encoding LT and F4 in the feces (Figs. 3 and 4), with no statistically difference in prevalence between these genes and those encoding STa and STb (*p* = 1). These results indicate that the genes encoding LT and F4 were derived exclusively from the challenge and were considered as marker genes for the challenge strain (F4-positive ETEC). Thus, at d1, a significant fecal presence of putative F4-positive ETEC was observed in the challenged groups compared with the unchallenged groups (*p <* 0.0001). In addition, no fecal presence of putative F4-positive ETEC was observed in the unchallenged groups at d1 and throughout the experiment.

After 3 days of successive oral treatment with CS (d4), a significant reduction in the prevalence of fecal presence of putative F4-positive ETEC was observed in the challenged treated group compared to the challenged untreated group (*p <* 0.0001) (Figs. 3 and 4). Similarly, a significant reduction in the prevalence of putative F4-negative ETEC in fecal samples was observed in the unchallenged treated group compared with the unchallenged untreated group (*p <* 0.0001) (Figs. 1 and 2).

From d8, corresponding to 2 days after CS oral treatment discontinuation, the genes encoding for LT and F4 were not detected in the feces of any challenged treated pigs although no difference was observed in the prevalence of putative F4-positive ETEC in fecal samples between the challenged treated and the challenged untreated group (*p* = 0.07) (Figs. 3 and 4). At d8 and d13, no difference was observed in the prevalence of putative F4-negative ETEC in fecal samples between the 2 unchallenged groups (*p =* 0.07) (Figs. 1 and 2).

At d36, which corresponds to 30 days after CS oral treatment discontinuation, a significant reduction in the prevalence of fecal presence of putative F4-negative ETEC was observed in the challenged untreated group compared to the unchallenged untreated group as demonstrated by the presence of the gene encoding STa (*p <* 0.001) (Fig. 1). At d13 and d36, no putative F4-positive ETEC in fecal samples was observed in either challenged group.

**Fig. 5:**
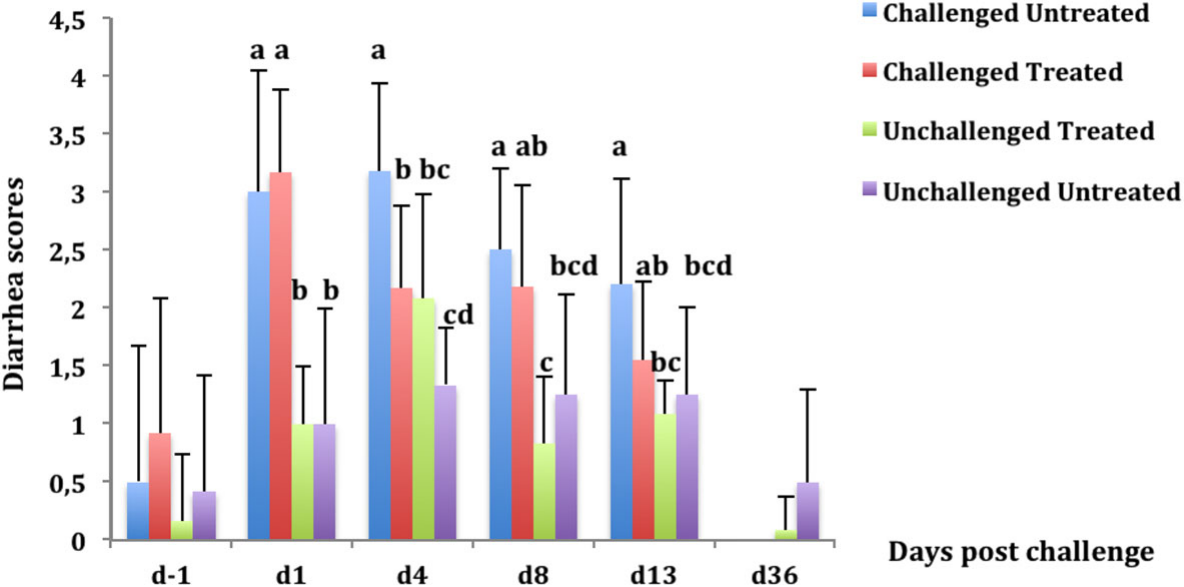
Evolution of diarrhea scores (mean ± standard deviation [SD]) in pigs challenged or not with an ETEC: F4 strain. Challenge was performed at d0 and treatment with colistin sulfate at the dose of 50, 000 IU/kg was started at d1 (24 h post challenge) and continued twice daily for 5 days. For each sampling time, means with different letters on a given day are statistically different. At d-1 and d36, there was no significant difference between groups

Prior to the bacterial challenge (d-1), no difference was found between the 4 groups regarding diarrhea scores (*p =* 0.33) (Fig. 5). At d1, a significant increase in diarrhea score was observed in the challenged groups compared with the unchallenged groups (*p <* 0.0001). At d4, a significant reduction in diarrhea score was observed in the challenged treated group compared with the challenged untreated group (*p <* 0.0001). Moreover, this finding was associated with a significant reduction in prevalence of putative F4-positive ETEC in the fecal samples of the challenged treated group.

Starting from d8, no significant difference was observed between the challenged untreated and the challenged treated group with respect to diarrhea scores (Fig. 5).

Furthermore, all challenged and unchallenged pigs, had rectal temperatures ranging mainly between 38.75 and 39.55 °C before the challenge, and the oral challenge with ETEC: F4 did not result in an increase in rectal temperature of challenged piglets compared to the control groups (Additional file 1). In challenged pigs, some piglets developed hypothermia (36 °C) that was observed several days post challenge; this hypothermia was sometimes followed by death of the pig.

Mean rectal temperatures in the two challenged groups at d4 post challenge were significantly lower compared to those of the unchallenged groups (*p <* 0.001). Other than at d4, no difference was observed for other days between challenged and unchallenged groups regarding rectal temperatures (*p* > 0.15) (Additional file 1).

Mortality was noted only in the challenged groups. One pig in the challenged treated group died 2 days after the oral challenge, after it received a single oral dose of CS, and two pigs in the challenged untreated group died at 4 and 6 days after the challenge. All pigs died following presention of acute diarrhea and anorexia.

## Discussion

In this study, the fecal presence of genes encoding STa, STb, LT, F4 and F18 in pigs challenged with an ETEC: F4 strain was determined in order to follow the fecal ETEC population, as an indicator of oral CS treatment efficacy in experimental PWD. The presence of ETEC virulence genes was investigated in enriched fecal samples rather than in *E. coli* isolates, as in other studies [17]. Hence, we used the terminology “putative” to describe the F4-positive or F4-negative ETEC populations. Nevertheless, we consider that our method is specific, as it has been reported in several studies that STa, STb, LT, F4 and F18 were found only in *E. coli* [2, 13].

In the present study, close to 100% of pigs excreted putative F4-negative ETEC in the feces before the oral challenge. To our knowledge, our study is the first to report such a finding in clinically healthy pigs in the post-weaning period. In fact, other studies have associated the presence of isolates possessing STa and STb genes with clinical PWD in farm conditions [18–20]. Nevertheless, Casey and collaborators constructed ETEC strains expressing either STa or STb, and diarrhea was only demonstrated following the inoculation of piglets with the STa construct expressing the fimbriae F41 [21]. In the current study, we have shown that in controlled conditions (optimal temperature, good sanitation, biosecurity procedures), the presence of putative F4-negative ETEC in the intestine is not always associated with clinical PWD in pigs.

In our study, three successive days of oral CS treatment, d4, was associated with a significant reduction in the fecal presence of both the putative F4-positive ETEC population and of the putative F4-negative ETEC population. At the same time, there was a significant reduction in diarrhea scores in the challenged treated pigs. In addition, at d4, putative F4-positive ETEC were detected in 100% of pigs belonging to the challenged untreated group, at the same time as high diarrhea scores and of the greatest fecal shedding of ETEC: F4 bacteria was observed in this group, as previously described [10]. These findings highlight the primary role of the F4-positive ETEC population in the occurrence of clinical PWD symptoms in our study.

In the current study, we noted that pigs of the challenged groups did not develop a febrile response in the days that followed the oral challenge. On the other hand, it has been shown that the maximum increase in the rectal temperature of pigs challenged with an ETEC: F4 strain was observed at 6 and 12 h post-challenge [22]. However, in our study, rectal temperatures were not taken during the hours that followed challenge, hence we did not characterize the acute-phase response of challenged pigs.

Interestingly, after only 3 days (d4) of oral administration of CS at 50,000 IU/kg bw, a significant reduction in both the F4-positive and F4-negative ETEC populations as well as in diarrhea scores was observed in the challenged treated group. Indeed, this duration of CS treatment is used in several countries compared to the period of 5 days [23]. We consider that our finding is important, having observed an association between CS treatment duration and CS pressure selection on the *E. coli* population during the treatment of pigs in the experimental PWD model [10]. Nevertheless, the effectiveness of the period, 3 or 5 days, of CS oral treatment in reducing the fecal excretion of F4-positive ETEC and its role in CS resistance *E. coli* amplification, needs to be confirmed in farm conditions with more animals and in the presence of other infection pressures. Such clinical data will be very relevant in the determination of oral CS effectiveness in PWD treatment and help in the re-evaluation of colistin treatment in pigs as undertaken by some regulatory agencies such as the European Medicines Agency (EMA) [24].

Even though pigs were clinically healthy when they excreted F4-negative ETEC before the challenge, we cannot exclude the role of this population in the potentiation of F4-positive ETEC isolates in the development of PWD. In fact, a tendency in the reduction of prevalence of genes encoding for STb prevalence in fecal samples was observed in association with a reduction in diarrhea scores in the challenged treated pigs. Moreover, it is recognized that PWD is a multifactorial disease, for which the many factors necessary to induce diarrhea has not yet been fully identified [25].

In the present study, starting from day two after the termination of CS oral treatment (d8) and up to the end of the experiment, no fecal presence of F4-positive ETEC was detected in the challenged treated group. On the other hand, a significant reduction in the F4-positive ETEC population and diarrhea scores was observed in the challenged untreated group, even in the absence of CS treatment. These findings could be explained by the effective immune response against ETEC: F4 in the challenged groups. Indeed, several studies have shown that oral immunization of weaned piglets with F4 fimbriae induced a systemic F4-specific antibody response and an increase in mucosal F4-specific antibody (IgA, IgM, IgG) in intestinal tissues [26–28]. On the other hand, after the termination of CS oral treatment, the fecal F4-negative ETEC population reappeared in the unchallenged treated group to the same extent as observed in the unchallenged untreated group. This finding confirmed the role of the immune response following the oral challenge with ETEC: F4 in a long-lasting protection of pigs against this pathogen.

In our study, 30 days after the termination of CS treatment (d36), pigs in the four experimental groups showed a fecal presence of a F4-negative ETEC population, with a lower prevalence than observed at d1, but usually without clinical symptoms of PWD. Once again, these findings should be considered when determining the cause of diarrhea in pigs using PCR to monitor ETEC virulence genes. Hence, the fecal presence of F4-negative ETEC in diarrheal pigs should not confirm the ETEC etiology of the PWD. Thus, this finding contributes to avoiding the use of antimicrobials to treat viral or parasitic diarrhea in the post-weaning period.

## Conclusion

The use of enriched fecal samples to investigate the fecal presence of ETEC enterotoxin as well as F4 and F18 genes by PCR, gave information about *E. coli* virulence profiles found in the gut of weaned pigs.

Under controlled conditions, CS oral treatment significantly reduced both the fecal F4-positive and F4-negative ETEC populations in treated groups, and this finding was associated with a significant reduction in diarrhea scores. Furthermore, the fecal presence of F4-negative ETEC was not associated with clinical PWD in pigs. A long-term field trial investigation with more animals would be helpful to confirm the effect of CS on fecal ETEC populations in farm conditions.

## Additional file

Additional file 1: Rectal temperatures (mean ± standard deviation [SD]) of weaned pigs challenged or not with ETEC: F4. Challenge was performed at d0 and treatment with colistin sulfate at the dose of 50,000 IU/kg was started at d1 (24 h post challenge) and administered twice daily for 5 days. (PPTX 33 kb)

## Abbreviations

ANOVA: Analysis of variance
BPW: Buffered peptone water
bw: Body weight
CFU: Colony forming units
CS: Colistin sulfate
DNA: Deoxyribonucleic acid
EMA: European Medicines Agency
ETEC: Enterotoxigenic *Escherichia coli*
F18: Fimbriae F18
F4 (K88): Fimbriae F4
Ig: Immunoglobulin
IU: International unit
LB: Luria-Bertani
LT: Heat-labile enterotoxin
Nal^R^: Nalidixic acid-resistant
PCR: Polymerase chain reaction
PWD: Post-weaning diarrhea
RFLP: Restriction fragment length polymorphism
SD: Standard deviation
STa: Heat-stable enterotoxin a
STb: Heat-stable enterotoxin b
