# Supplementary material for: The fecal presence of enterotoxin and F4 genes as an indicator of efficacy of treatment with colistin sulfate in pigs
Source: BMC Microbiol. 2017 Jan 5;17:6. doi: 10.1186/s12866-016-0915-0 (PMC5217267; doi:10.1186/s12866-016-0915-0)
Supplement: Additional file 1: — Rectal temperatures (mean ± standard deviation [SD]) of weaned pigs challenged or not with ETEC: F4. Challenge was performed at d0 and treatment with colistin sulfate at the dose of 50,000 IU/kg was started at d1 (24 h post challenge) and administered twice daily for 5 days. (PPTX 33 kb) [file 12866_2016_915_MOESM1_ESM.pptx]

## Slide 1
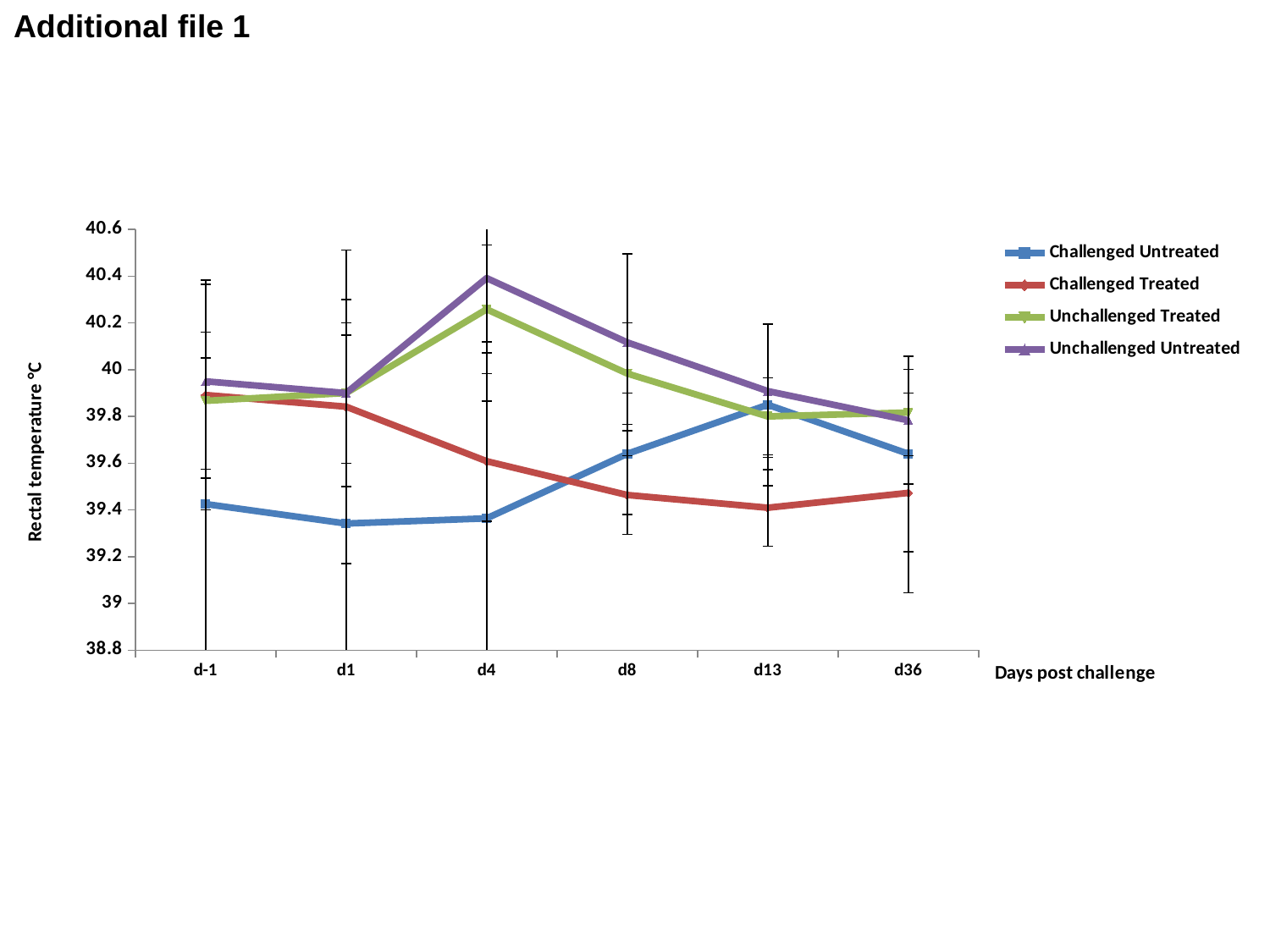

Additional file 1
### Chart
| Category | Challenged Untreated | Challenged Treated | Unchallenged Treated | Unchallenged Untreated |
|---|---|---|---|---|
| d-1 | 39.425 | 39.89166666666661 | 39.86666666666661 | 39.95 |
| d1 | 39.3416666666666 | 39.8416666666666 | 39.9 | 39.9 |
| d4 | 39.36363636363634 | 39.60833333333333 | 40.25833333333333 | 40.39166666666661 |
| d8 | 39.64 | 39.46363636363634 | 39.98333333333333 | 40.11666666666661 |
| d13 | 39.85 | 39.40909090909091 | 39.8 | 39.90833333333333 |
| d36 | 39.64 | 39.47272727272727 | 39.81666666666661 | 39.78333333333333 |
